# Human *Trypanosoma cruzi* Infection and Seropositivity in Dogs, Mexico

**DOI:** 10.3201/eid1204.050450

**Published:** 2006-04

**Authors:** Jose G. Estrada-Franco, Vandanajay Bhatia, Hector Diaz-Albiter, Laucel Ochoa-Garcia, Alberto Barbabosa, Juan C. Vazquez-Chagoyan, Miguel A. Martinez-Perez, Carmen Guzman-Bracho, Nisha Garg

**Affiliations:** * University of Texas Medical Branch, Galveston, Texas, USA;; † Instituto de Salud del Estado de Mexico, Toluca, Mexico;; ‡ Universidad Autonoma del Estado de Mexico, Toluca, Mexico;; § Instituto de Diagnostico y Referencia Epidemiologicos Secretaría de Salud, Mexico City, Mexico

**Keywords:** Mexico, Trypanosoma cruzi, seroprevalence, humans, dogs, research

## Abstract

We used 5 diagnostic tests in a cross-sectional investigation of the prevalence of *Trypanosoma cruzi* in Tejupilco municipality, State of Mexico, Mexico. Our findings showed a substantial prevalence of immunoglobulin G (IgG) and IgM antibodies to *T*. *cruzi* in human (n = 293, IgG 2.05%, IgM 5.5%, both 7.1%) and dog (n = 114, IgG 15.8%, IgM 11.4%, both 21%) populations. We also found antibodies to *T*. *cruzi* (n = 80, IgG 10%, IgM 15%, both 17.5%) in dogs from Toluca, an area previously considered free of *T*. *cruzi*. Our data demonstrate the need for active epidemiologic surveillance programs in these regions. A direct correlation (*r*^2^ = 0.955) of seropositivity between humans and dogs suggests that seroanalysis in dogs may help identify the human prevalence of *T*. *cruzi* infection in these areas.

## Abstract 2

Seroanalysis of parasite circulation in dogs can help identify *T*. *cruzi* infection in humans.

*Trypanosoma cruzi*, which causes Chagas disease, affects ≈17.4 million people in the Western Hemisphere (*1*). The first case of human infection with *T*. *cruzi* in Mexico was reported in 1936 (*2*). A national serosurvey from 1987 to 1989 reported a seroprevalence of 1.6% (≈1.6 million people) and widespread *T*. *cruzi* infection in the inhabitants of 23 of the 32 provinces of Mexico (*3**,**4*). A similar prevalence of *T*. *cruzi*–specific antibodies (1.5%) was observed in national blood bank repositories (*5*). Rural Mexican villages were confirmed as endemic zones for *T. cruzi*. Other investigators reported <20% seropositivity in inhabitants of rural areas south of the Tropic of Cancer (*4**,**6**,**7*).

In 1992, the State of Mexico was documented to be free of *T*. *cruzi* (n = 2,800 seropositive, <0.2%) (*3*). Another survey of 3,300 blood donors in Mexico City identified a seropositive rate of 0.3% (*8*), and many of these donors had no history of traveling to disease-endemic areas. Other studies from 1998 to 2000 reported acute cases of *T. cruzi* infection and seropositivity among inhabitants of the State of Mexico (9 and C. Guzman-Bracho, unpub. data).

Circulation of *T*. *cruzi* is maintained by the interaction of bloodsucking triatomines with humans and reservoir animal hosts (*1*). Of the 31 triatomine species identified in Mexico, *Triatoma barberi*, *Triatoma dimidiata*, and *Triatoma pallidipennis* have the highest vectorial activity in central and southern Mexico (*4**,**10**,**11*). An entomologic survey in the spring of 2001 documented widespread distribution of *T*. *pallidipennis* in the southern part of the State of Mexico (infestation index 9.9%, density index 2.7%–3.0%) and suggested that active transmission of *T*. *cruzi* may occur (*12*).

Dogs are considered important in the dynamics of *T*. *cruzi* infection of triatomines and transmission within human dwellings (*1**,**13**,**14*). Seropositive domestic and stray dogs have been found in some states of Mexico (*15**–**17*). However, the prevalence of *T*. *cruzi* in dogs and the role of these reservoir animals in parasite transmission in the State of Mexico have not been determined.

In this study, we report the seroprevalence of *T. cruzi* among persons and dogs in the villages in the southern part of the State of Mexico and discuss the potential diagnostic meaning of seropositivity in dogs for identifying seroprevalence in humans. We also present data suggesting the likelihood of *T*. *cruzi* transmission in Toluca. Our observations emphasize that relevant health agencies need to conduct active epidemiologic surveillance programs and implement vector control strategies in the State of Mexico.

## Materials and Methods

### Parasites

*T*. *cruzi* epimastigotes were cultivated as previously described (*18*). Epimastigote (Mexican isolates) antigen extract was used for the serologic tests conducted at the Instituto de Diagnostico y Referencia Epidemiologicos Secretaría de Salud (InDRE) Mexico City. Trypomastigotes (SylvioX10/4) were propagated in monolayers of C2C12 cells (*19*) and used in the studies at the University of Texas Medical Branch (UTMB) in Galveston.

### Study Area, Population, and Sample Collection

**Figure 1:**
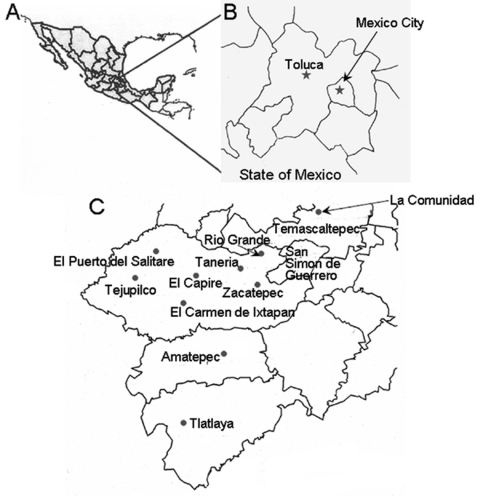
Study site in Mexico. A) Country of Mexico. B) State of Mexico. C) Southern part of the State of Mexico. Shown are the municipalities and villages in the State of Mexico where epidemiologic serosurveys were conducted.

This study was conducted in southern villages of the Tejupilco municipality, State of Mexico (Figure 1). The area has seasonal climate variations (dry season from November through May and rainy season from June through October). The population is primarily indigenous, and the main occupations are agriculture and livestock production. Migration occurs among the men to cities in Mexico and to northern border regions near the United States.

For the human serosurvey (N = 356), we selected 5 villages (altitude range 1,090–1,730 m) where triatomine infestation was reported by the Instituto de Salud del Estado de Mexico (ISEM) in >50% of the households. For comparison, we also selected La Comunidad village (altitude 2,500 m) in the same area. Since this study focused on evaluating active *T*. *cruzi* transmission, most test samples (>94%) were from children (age range 2–15 years) with a sex distribution consistent with the regional and national census. Sample randomization was controlled by using EpiInfo version 3.3.2 (Centers for Disease Control and Prevention, Atlanta, GA, USA). Oral informed consent was obtained from adults and parents of minors enrolled in the study. Trained ISEM personnel performed venipuncture to obtain blood samples. The study was reviewed and approved by the human subjects committees at ISEM and UTMB.

Dog serum samples were collected in Toluca and the villages selected for human screening. Toluca, the capital of the State of Mexico (altitude 2,680 m, average temperature 15°C, range 5°C–24°C) is considered free of vectorial *T*. *cruzi* transmission because triatomines (with or without *T*. *cruzi*) have never been documented in the area, and triatomines are believed not to proliferate at altitudes >2,500 m (*3*,*4*). Serum samples from 20 healthy dogs from an animal clinic in Hamburg, Germany, were used as negative controls. All animal experiments were reviewed and approved by the animal welfare committee at ISEM.

### Serologic Analysis

At UTMB, human and dog serum samples were screened for antibodies to *T*. *cruzi* by enzyme-linked immunosorbent assay (ELISA) as previously described (*20**,**21*). All samples and controls were assayed in triplicate in at least 2 independent experiments. Seropositive samples were confirmed by immunofluorescence flow cytometry (IFC) (*21*), and data were expressed as the relative percentage of positively fluorescent parasites.

At InDRE, serum samples were analyzed for immunoglobulin G (IgG) antibodies to *T*. *cruzi* by ELISA, an indirect hemagglutination (IHA) test, and an indirect immunofluorescence (IIF) assay. For the ELISA, 96-well, flat-bottomed plates were UV irradiated, incubated for 1 h at 37°C with epimastigote antigen extract, and blocked with 50 μL Tris-buffered saline, 0.1% Tween 20, and 5% nonfat dry milk. Plates were incubated at 37°C with 50 μL of each test serum sample (1:50 dilution) for 2 h, horseradish peroxidase–conjugated IgG (1:50 dilution) for 1 h, and substrate (o-phenylenediamine) for 20 min. The reaction was stopped by adding 2 N H_2_SO_4_, and the optical density (OD) was read at 490 nm (*22*). The IHA and IIF assays were performed with 4-fold serial dilutions of serum samples (range 1:8–1:128) (*5*), and samples were considered seropositive when a strong signal was obtained at a dilution >1:16. Epimastigote antigen extract was not used to determine IgM seropositivity for *T*. *cruzi* in this study because it has shown limited sensitivity (*23**,**24*).

### Statistical Analysis

Significance (p<0.05) was determined with the Student *t* test and validated with the Fisher exact test. The level of agreement for serologic data from 5 tests conducted at UTMB and InDRE was assessed as previously described (*25*).

## Results

### Standardization of Serologic Assays

**Figure 2:**
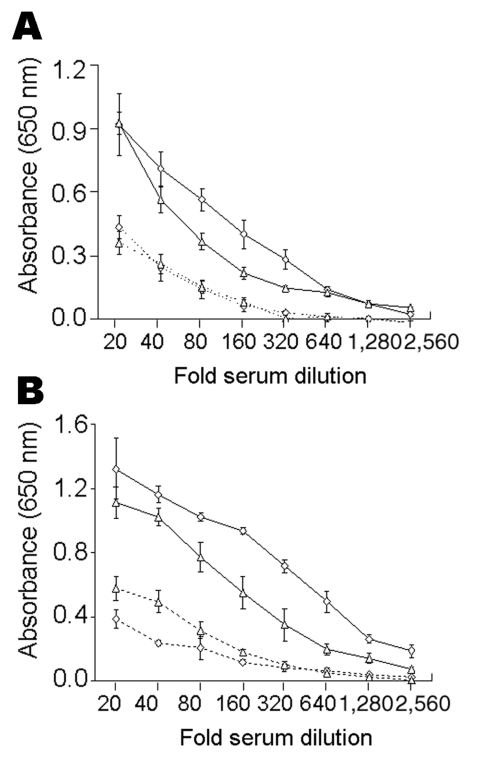
Serum titration curves of enzyme-linked immunosorbent assays comparing absorbance values for serial dilutions of pooled positive (solid lines) and negative (dashed lines) control sera from humans (A) and dogs (B). Absorbance values for immunoglobulin G (IgG) and IgM antibodies to *Trypanosoma cruzi* are represented by triangles and diamonds, respectively.

Results of the trypomastigote-based ELISAs for IgG and IgM antibodies in positive and negative samples are shown in Figure 2. Variations in reactivity of negative and positive sera in different experiments and the same experiment ranged from 3% to 12%. The highest signal-to-noise ratios between positive and negative controls from humans (IgG 4.0, 6.5, 9.1 and IgM 3.6, 5.5, 11.3) were obtained at dilutions of 1:80, 1:160, and 1:320, respectively (Figure 2A). Thirty-five serum samples and pooled negative samples (1:100 dilution) were analyzed by ELISA (Figure 2A). Mean cutoff OD values were 0.194 for IgG and 0.270 for IgM.

The highest signal-to-noise ratios by ELISA between positive and negative controls from dogs (IgG 4.3, 7.9, 8.8 and IgM 2.4, 3.0, 3.5) were obtained at dilutions of 1:80, 1:160, and 1:320, respectively (Figure 2B). Cutoff OD values of 0.288 for IgG and 0.219 for IgM were obtained with serum samples from uninfected dogs in Mexico and Germany.

**Figure 3:**
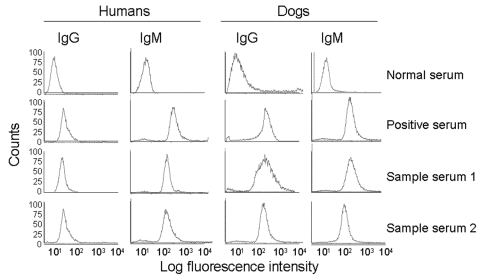
Detection of antibodies to *Trypanosoma cruzi* by immunofluorescence flow cytometry. Fluorescein isothiocyanate fluorescence intensities for *T*. *cruzi*–specific immunoglobulin G (IgG) and IgM antibodies in human and dog serum samples are shown. Background staining with normal serum, positive staining with chronic serum, and representative staining with 2 of the test serum samples are shown.

All serum samples were analyzed at a 1:100 dilution by ELISA and IFC. The highest signal-to-noise ratio for detection of antibody to trypomastigote surface antigens by IFC was obtained with 5 × 10^5^ parasites/reaction, which was also reported in other studies (*21**,**26*). Positive and negative control peaks were distinguishable: >90% of trypomastigotes incubated with negative sera (1:100 dilution) had a fluorescence intensity (LFI) <10 and 50%–98% of trypomastigotes incubated with positive sera (1:100 dilution) had an LFI of 10^2^–10^3^ (Figure 3).

### Seroprevalence of *T*. *cruzi*–specific Antibodies in Humans

At UTMB, we identified 9 seropositive persons (mean seroprevalence 3.1%, range 0%–21%) from the villages of Tejupilco municipality. The mean OD value, after subtracting for background, for the seropositive population was 0.213, and the highest value was 0.419. IgG seropositivity was similarly distributed among men and women (55% vs. 45%). All seropositive samples identified by ELISA were positive for *T*. *cruzi*–specific IgG by IFC. Approximately 73% of the trypomastigotes (range 62%–91%) incubated with positive serum samples had an LFI of 10 to 500. Trypomastigotes (>98%) incubated with negative serum samples had an LFI <10 (Figure 3). The results of ELISA and IFC for detection of *T*. *cruzi*–specific antibodies showed 100% agreement. *T*. *cruzi*–specific IgG was also detected by ELISA, IIF, and IHA in a blind study at InDRE. This study identified 7 seropositive human patients, of whom 6 were positive by UTMB tests (Table 1). The maximal percentage seropositivity was identified in El Carmen Ixtapan and El Puerto del Salitre, which are located at low altitudes (Table 2). All 63 persons tested in La Comunidad were seronegative (Table 2).

**Table 1:** Prevalence of immunoglobulin G (IgG) antibodies to *Trypanosoma cruzi* in persons in southern area of the State of Mexico*†

No. positive test results‡	Total screened		Tejupilco		Temascaltepec	
	No. (%)	% of population	No. (%)	% of population	No. (%)	% of population
0	321 (90.1)		268 (91.5)		55 (87.31)	
1	22 (6.2)		14 (4.78)		6 (9.52)	
2	7 (2.0)	98.3	5 (1.7)	97.95	2 (3.17)	100
3	**1 (0.28)**		**1 (0.35)**		0	
4	**2 (0.56)**		**2 (0.68)**		0	
5	**3 (0.85)**	**1.7**	**3 (1.02)**	**2.05**	0	0.0
Total	356 (100)		293 (100)		63 (100)	

*Samples positive in >3 tests were considered seropositive (shown in **boldface**). †All except 2 participants seropositive for IgG antibodies by >2 tests were 5–18 y of age. ‡Level of agreement for serologic data for 5 tests: concordance level 98.2; κ coefficient 0.618; 95% confidence interval 0.622–0.714.

**Table 2:** Prevalence of antibodies to *Trypanosoma cruzi* in persons in southern area of the State of Mexico*

Municipality	Village	Altitude (m)	No. screened	Seropositivity,† no. (%)		
				IgG positive	IgM positive‡	IgG and IgM positive
Tejupilco	El Carmen Ixtapan	1,091	16	1 (6.3)	3 (18.7)	4 (25.0)
	El Puerto del Salitre	1,268	29	3 (10.3)	ND	3 (10.3)
	Zacatepec	1,311	200	2 (1.0)	10 (5.0)	11 (5.5)
	Rio Grande	1,554	3	0	ND	0
	Tenería	1,730	45	0	3 (6.6)	3 (6.6)
Subtotal			293	6 (2.05)	16 (5.5)	21 (7.1)
Temascaltepec	La Cominidad	2,500	63	0	1 (1.6)	1 (1.6)

*IgG, immunoglobulin G. †p<0.001 for IgG, IgM, and IgG plus IgM seropositivity. ‡All IgM-seropositive persons were 4–13 y of age. ND, not determined.

Our data showed that 16 (5.5%) of 293 persons in Tejupilco were seropositive for IgM antibodies to *T*. *cruzi* (Table 2). The prevalence of IgM antibodies was higher in female than in male patients (64% vs. 36%). All serum samples positive by ELISA for IgM antibodies were also positive by IFC (50%–93% of the parasites with an LFI of 10^2^–10^3^) (Figure 3). The overall prevalence of *T*. *cruzi*–specific antibodies (IgG and IgM) in persons in Tejupilco was 7.1% (21/293) (Table 2).

### Seroprevalence of *T. cruzi*–specific Antibodies in Dogs

**Figure 4:**
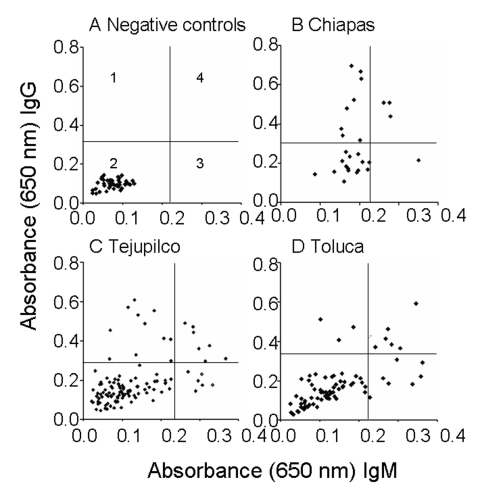
Distribution of immunoglobulin G (IgG) and IgM antibodies to *Trypanosoma cruzi* in dogs. An enzyme-linked immunosorbent assay was used to detect antibodies in dogs in Tejupilco (C) and Toluca (D) in the State of Mexico. Negative controls are shown in A. Seroanalysis of dogs from Chiapas, a *T*. *cruzi*–endemic zone, is shown in B. The quadrants in A indicate the following: 1, IgG positive; 2, IgG and IgM negative; 3, IgM positive; 4, IgG and IgM positive.

We used dog serum samples from Chiapas, where *T*. *cruzi* infection and transmission were reportedly endemic (*7*), as positive controls. These dogs had a seropositivity of 39.3% for IgG and 14.3% for IgM (Table 3 and Figure 4B). Dogs in Tejupilco had antibodies to *T*. *cruzi* (IgG 15.8%, IgM 11.4%, IgG and IgM 21.0%) (Table 3). A total of 6.1% of the dogs from Tejupilco were positive for both IgG and IgM (Figure 4C), and no sex-related differences in prevalence of parasite-specific antibodies were observed. IgG seropositivity increased with age, with the highest seroprevalence in dogs 3–6 years of age. All samples seropositive by ELISA were seropositive by IFC. A total of 57% to 94% of the parasites showed IgG-specific staining (LFI 10^2^–10^4^), and 86%–98% showed IgM-specific staining (LFI 100 to 4 × 10^3^) (Figure 3). Samples seropositive for IgG were confirmed by IHA (data not shown). None of the serum samples from dogs in northern villages (Apaxco, Hueypoxtla, Jaltenco, and Nextlalpan) in the State of Mexico or the German veterinary clinic (Figure 4A) had *T*. *cruzi*–specific antibodies. This result demonstrated the specificity and sensitivity of the assays used. Pairwise linear analysis showed a positive correlation of IgG seropositivity in dogs and humans in study area (*r*^2^ = 0.955). Parasite-specific antibodies (IgG 10%, IgM 15%, IgG and IgM 17.5%) were detected in dogs from Toluca (Table 3 and Figure 4D), a region previously considered free of *T*. *cruzi* infection.

**Table 3:** Prevalence of antibodies to *Trypanosoma cruzi* in dogs in the southern area of the State of Mexico

Municipality	Village	No. screened	Seropositivity,* no. (%)		
			IgG positive†	IgM positive‡	IgG and IgM positive
Tejupilco	El Carmen Ixtapan	16	5 (31.3)	0	5 (31.3)
	Rincon del Carmen	42	10 (23.8)	13 (30.9)	16 (38.0)
	Rio Grande	24	1 (4.2)	0	1 (4.2)
	Tejupilco	10	1 (10.0)	0	1 (10.0)
	Zacatapec	22	1 (4.5)	0	1 (4.5)
Subtotal		114	18 (15.8)	13 (11.4)	24 (21.0)
Toluca		80	8 (10.0)	12 (15.0)	14 (17.5)
Northern area§		24	0	0	0
Chiapas		28	11 (39.3)	4 (14.3)	12 (42.8)

*IgG, immunoglobulin G. p<0.001 for IgG, IgM, and IgG plus IgM seropositivity. †IgG-seropositive dogs were 8 mo to 6 y of age; 85% were >2 y of age. ‡IgM-seropositive dogs were 4 months to 6 years of age; a similar distribution was observed in all age groups. §Northern villages of Apaxco, Hueypoxita, Jaltenco, and Nextlalpan were included in this group.

## Discussion

We detected *T*. *cruzi*–specific IgG and IgM in 7.1% of persons and 21.0% of dogs in Tejupilco. In addition, we observed an IgG and IgM seroprevalence of 17.5% in dogs in Toluca, which was previously reported to be free of *T. cruzi* infection. Epimastigote and trypomastigote antigens and 5 different tests were used to determine IgG seropositivity in selected areas. Nine of 293 IgG-positive patients were identified by trypomastigote-based tests, of whom 6 were also identified by >2 of the epimastigote-based tests at InDRE. Low IgG seropositivity in the InDRE survey might have occurred because epitopes shared by epimastigote and trypomastigote forms are intracellular antigens, whereas the IgG antibodies are specific for trypomastigote surface antigens (*27*). Alternatively, low seropositivity may be attributed to different parasite strains used for serologic tests at the 2 institutes. A positive correlation between IgG seropositivity in humans and dogs (*r*^2^ = 0.955) implies that dogs help identify or monitor seroprevalence in these populations.

Serologic analysis has been reported to be limited in acute infections with *T. cruzi* (*23**,**24*). With trypomastigote antigens, we detected an IgM seroprevalence of 5.5% in humans, thus demonstrating that a robust *T*. *cruzi*–specific IgM response is mounted by humans. In addition, most of the IgM-positive persons were IgG negative. These data, along with the observation of high IgM seropositivity (11.4%) in dogs from the same area, suggest the occurrence of acute *T*. *cruzi* infection in rural villages in the State of Mexico. This view is supported by Wickner et al., who reported polymerase chain reaction–based detection of *T*. *cruzi* in blood samples from patients with acute infection identified as IgM seropositive by ELISA (agreement 94%) (*28*).

Our study identified seropositive patients (7.1%) and high seroprevalence (21.0%) in dogs from southern villages in the State of Mexico, which has a low altitude (<1,700 m) and warm temperatures. Nearly all inhabitants screened in the study had dogs that lived near their owners in small quarters, and we observed a correlation between seropositivity in dogs and humans in these communities.

Previous reports showed infestation with *T*. *barberi* and *T*. *pallidipennis* at low altitudes (<2,000 m) in all areas of Mexico (*4*) and inside and around houses in the southern part of the State of Mexico (*12*). Dogs provide frequent blood meals for *T*. *barberi* and *T*. *pallidipennis* and may acquire *T*. *cruzi* infection by ingesting infected triatomines. We surmise that the active transmission of *T*. *cruzi* occurs in the southern part of the State of Mexico, and the presence of *T*. *cruzi* in dogs and insect vectors can help determine the prevalence of *T*. *cruzi* infection in humans. Thus, low altitudes and warm temperatures may sustain vectorial activity and *T*. *cruzi* transmission in southern Mexico. Several observations support our hypothesis. First, dogs maintain parasitemia long after infection (*29*) and are the preferred source of blood meals for *Triatoma infestans* (*30*). Second, the prevalence rate of infective *T*. *infestans* in a household increases with the number of infected dogs in the vicinity (*13*). In accordance with the increase in infected insects, the seroprevalence of infected adults doubled in households with 1 to 2 infected dogs (*14*). Third, *T*. *cruzi*–specific antibodies have been identified in humans (4%) and dogs (10%) in rural villages of Puebla, Mexico, where active vectorial transmission, shown by a high dispersion area index (55%) and colonization index (40%), was also noted (*17*).

We observed a high seroprevalence of IgG and IgM antibodies (17.5%) in dogs from Toluca. Additional studies would determine whether changes in behavior and localization of triatomines at higher altitudes may lead to *T*. *cruzi* infection in dogs in Toluca. Alternatively, a high rate of migration from endemic to nonendemic zones exists in Mexico. At institutional blood banks, ≈40% of donors reported to be permanent residents of Mexico City were born in other states of Mexico (C. Guzman-Bracho, unpub. data). These immigrants bring their domestic animals with them, and thus may inadvertently contribute to the spread of *T*. *cruzi* infection. Our detection of *T. cruzi* in dogs from Toluca suggests that this city and others in Mexico located at high altitudes may not be free of *T*. *cruzi* infection.

The seroprevalence of 21.0% (IgG and IgM) in dogs and the observed vectorial activity in these areas suggest that dogs may be domestic reservoir hosts and help maintain human transmission of *T*. *cruzi*. Our observations emphasize the importance of active epidemiologic surveillance programs throughout Mexico and implementation of sound vector control strategies in disease-endemic areas.
